# Management of an extrasphincteric fistula in an HIV-positive patient by using fibrin glue: a case report with tips and tricks

**DOI:** 10.1186/1471-230X-10-18

**Published:** 2010-02-14

**Authors:** Theodossis S Papavramidis, Ioannis Pliakos, Dimitra Charpidou, George Petalotis, Panagiotis Kollaras, Konstantinos Sapalidis, Isaak Kesisoglou, Spiros T Papavramidis

**Affiliations:** 1 3rd Department of Surgery, AHEPA University Hospital, Aristotle University of Thessaloniki, Thessaloniki, Greece; 2 Department of Radiology, AHEPA University Hospital, Aristotle University of Thessaloniki, Thessaloniki, Greece; 3 1st Department of Internal Medicine, AHEPA University Hospital, Aristotle University of Thessaloniki, Thessaloniki, Greece

## Abstract

**Background:**

Individuals with impaired immunity are at higher risk of perianal diseases. Concerning complex anal fistulas impaired healing and complication rates are also higher. Definitive treatment of a fistula aims controlling the purulent discharge and prevents its recurrence. It depends mainly on the trajectory of the fistula and the underlying disease.

We present a case of a HIV-positive patient with a complex extrasphincteric anal fistula who was treated successfully with fibrin glue application. We further, discuss tips and tricks when applying fibrin glue as plugging material in complex anal fistulas.

**Case presentation:**

A sixty-one-year-old HIV-positive male referred to us for warts and extrasphincteric fistula. Because of the patients' immunological status, we opted against surgery and recommended fibrin glue plugging. The patient was discharged the same day. A follow-up examination was performed 5 days after the initial fibrin glue application showing that the fistula canal was obstructed. Three months and a year post-intervention the fistula tract remains closed.

**Conclusion:**

The best treatment for a disease gives at least the same result with the other treatments with minimised risk for the life of the patient and minimal application effort. Conservative closure of fistula with fibrin plugging is simple, safe and with less morbidity than surgery. Our patient was successfully treated without endangering his life despite his precarious medical state. Not everybody believes in the effectiveness of fibrin glue application, however we consider this solution in cases of complex fistulas at least as primary procedure in special populations such as the immunosupressed.

## Background

Individuals with diseases that reduce the body's immunity -such as AIDS or cancer- are at a higher risk of anal abscess and fistula formation [1]. However, despite the higher incidence of fistula formation, no differences were observed concerning the healing time in simple fistulas [2]. On the contrary, healing and complication rates were altered in complex fistulas [3].

In all individuals, definitive treatment of a fistula aims to control the purulent discharge and prevent its recurrence. Treatment depends mainly on the trajectory of the fistula and the underlying disease leading to the fistula formation [4,5]. The standard treatments that can be used are various, mostly surgical in nature, including fistulotomy, excision of the external fistula tract, closure of the internal opening, curettage, endorectal advancement flap, a cutting seton or immediate reconstruction [6].

Fibrin glue pluging is a method explored in recent years in various types of fistulas with great success [7-9]. Recently, fibrin glue has been applied in perianal fistulas with diverse success rates [4,5,10].

We present a special case of a HIV-positive patient with a complex extrasphincteric anal fistula who was treated successfully with fibrin glue application. We further, discuss tips and tricks when applying fibrin glue as plugging material in fistulas.

## Case presentation

**Figure 1:**
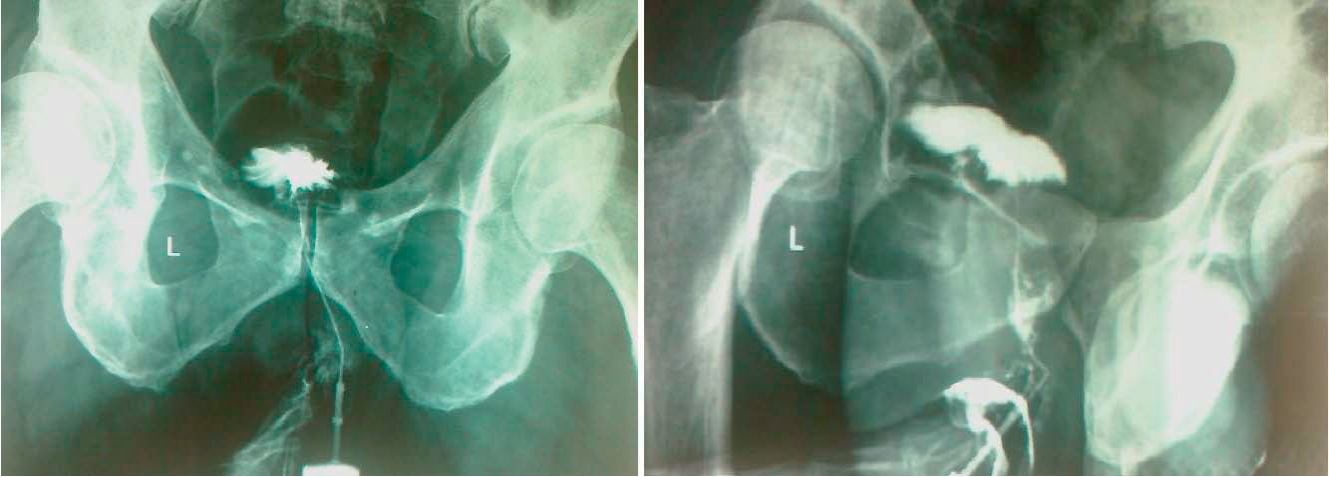
**Fistulography showing the tractus of the fistula**.

**Figure 2:**
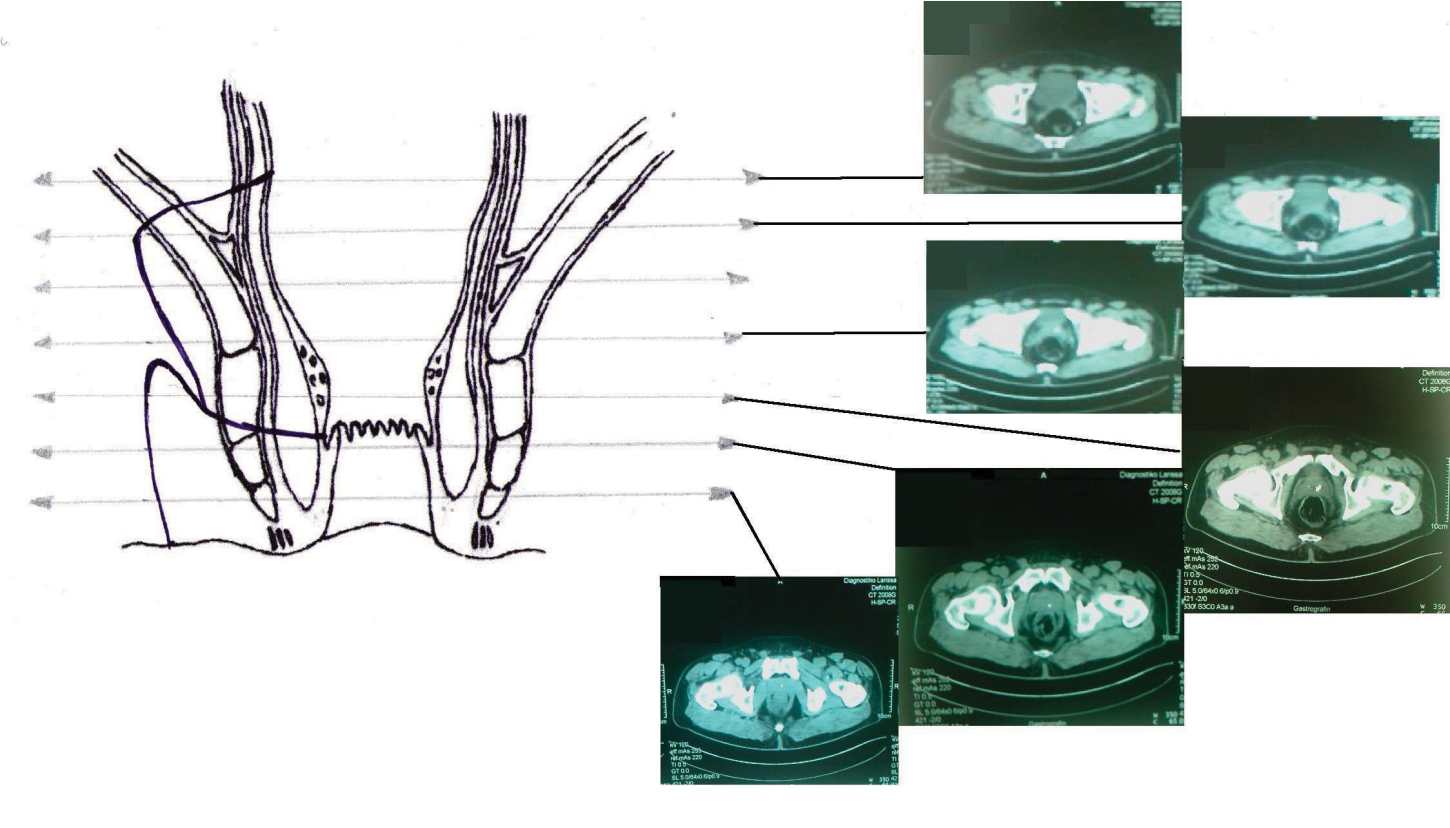
**Schematic presentation of the fistula in correlation with the MRI images**.

A sixty-one-year-old Caucasian male referred to our department suffering from anal pain and a combination of mucopurulent and stercoraceous discharges. The patient is HIV-positive with very low viral load, T4 577 c/ul, T8 1006 c/ul and T4/T8 = 1. During clinical examination, warts were found in the anal region. The external orifice of a fistula was in the right lateral wall of the internatal region. Prior any intervention, a fistulography (Figure 1) and a colonoscopy were performed as initial evaluation to help rule out any other disease process in the rectum. Fistulography revealed an extrasphincteric fistula with a 5 cm length and a 1.5 mm breadth. Colonoscopy didn't reveal any pathological findings. Because of the patients' immunological status, we opted against the surgical decision that required a colostomy formation, and instead recommended the closure of the fistula by using a fibrin glue plug. Prior fibrin glue application, on the same session, a new fistulography was performed, aiming to identify the internal orifice of the fistula and place a catheter proximal to this orifice, in order to infuse the glue (Starting from the internal orifice and coming out). During fistulography, we discovered the existence of a second anal fistula. As show in the schema (Figure 2), both fistulas shared a common internal orifice in the rectal wall. No acute purulence or inflammation of the fistulas was oberserved at the time of intervention. The two fistulas were managed separately. The anal fistula was managed by a seton-type prolen-0 stitch. Following the seton placement, we catheterized the external orifice of the extrasphincteric fistula, we were then able to place a catheter near the internal orifice of the fistula in order to infuse the fibrin glue. The fibrin sealant Beriplast P (Behring, Marburg, Germany) was used as fistula plug. The kit contains freeze-dried fibrinogen and aprotinin solution and freeze-dried thrombin and calcium chloride. These substances are mixed to form two components--sealer and thrombin solution--that are kept in two separate syringes. Local application of the components was accomplished under radioscopic vision via a catheter passed through fistula. The two components were injected with separate syringes, and simultaneous mixing at the catheter tip led to rapid coagulation and mechanically stable fibrin clot formation inside the fistula tract. Immediate post-interventional fistulography showed to evidence of fistula tract. The patient was discharged the same day. A follow-up examination was performed 5 days after the initial fibrin glue application. Catheterization of the internal orifice of the fistula showed that the fistula canal was closed. Three months and a year after the initial intervention a new radioscopic examination was scheduled proving that both anal fistulas were successfully closed.

## Discussion

Anal fistulas are divided into 4 categories, with the suprasphincteric category being the rarest and representing approximately 1% of the total number of fistulas. The present fistula additionally, enters in the category of "complex" fistulas after the definition given by Loungnarath et al [11]. By this definition "complex" annals fistulas are those in which treatment by simple fistulotomy would result in significant impairment of continence. This category generally consists of high transsphincteric, suprasphincteric and extrasphincteric fistulas. The treatment of complex anal fistulas is one of the most challenging aspects of coloproctology and colorectal surgery.

The complexity of the present case is aggravated by the fact that the patient is HIV-positive. Approximately 30% of patients with HIV develop anorectal abscesses and fistulas [3]. Anal fistulas in HIV-positive patients arise from the dentate line in similar locations to human immunodeficiency virus-negative patients [12]. Anorectal procedures (for anal condylomata, fistula in ano, hemorrhoids, and perirectal abscess) are the most common operations performed in HIV-patients. No prospective data are available on operative morbidity and mortality in HIV-positive and HIV-negative patients. In retrospectives studies, investigators found a high incidence of serious wound complications after anorectal surgery and urged non-operative treatment for most anorectal diseases in HIV-infected patients [2,3]. Although there is currently no scientific data on the prevalence of surgical complications among HIV patients compared to non-infected patients in colorectal surgery, it is more safely to avoid any type of major surgical treatment, as these patients have a weakened immune system. In this perspective, alternatives to conventional surgery were considered to the present patient.

Alternative to conventional surgery in complex anal fistulas are considered the use of fibrin glue (autologous or commercial) and the use of a biologic fistula plug (Surgisis^®^AFP™). Among those two choices we chose the first. Our choice was based on the fact that we had no experience with Surgisis and that all studies employing this material date less than 5 years, not permitting to evaluate the long-term results [13,14]. On the other hand, fibrin glue application is a safe, cheap, reproducible, pain-free procedure, which eliminates the possibility of anal incontinence and can be performed under local anaesthesia [15]. The fibrin glue application in the treatment of anal fistula is the guaranteed preservation of the sphincters, so it avoids the risk of incontinence, and creates minimal stress for the patient [4,5,10]. However, a final question arises -mainly theoretically- when coming to fibrin glue application: autologous or commercial? To our opinion commercial sealants have an advantage over autologous ones. Our impression seems to correlate well with the meta-analysis of Hammond et al [10]. According to this meta-analysis commercial sealants have been shown to bond more consistently -by up to 10 times stronger than autologous when compared in vitro-, take less time to prepare and do not require autologous blood transfusion. This was the reason why commercial fibrin glue was employed for plugging the anal fistula in the present case.

### Tips and tricks

We believe that in the present case we had a successful closure of the fistula due to tips and tricks that we have extracted from our experience both in simple anal fistulas and fibrin glue application. Patients' history and old hospitalizations have to be considered carefully in order to determine, if possible, the aetiology of the fistula and the predisposing factor. The visualization of fistulas' canal is of prime importance before any intervention -either surgical or not-. The preoperative evaluation includes colonoscopy, fistulography and MRI of the pelvis. In that way, the tract of the fistula is known before any intervention. Once the fistula is a "complex" one, then adequate treatment is applied after considering the general condition of the patient. In case of fibrin glue application, we perform no pre-operative bowel preparation. This strategy is in the same context as for all colonic operations in our department: intact colonic flora offers better healing [16]. Immediately, before the fibrin glue application we like to perform a new fistulography. Once the fistula tract is visualized we perform blunt curettage. Mechanical curettage stops when blood exits the external orifice of the fistula. After that, chemical cleansing with hydrogen peroxide follows aiming both in introducing oxygen radicals in the fistula (bactericide) and in achieving haemostasis. Following that, catheterization of the fistula tract is achieved with a catheter that has a slightly smaller in diameter than the fistula. A small quantity of radioopaque material is introduced into the catheter in order to assure that its tip is placed in proximity to the internal orifice. The fibrin glue is then prepared by an assistant and a plastic double-lumen Y-connector joins the two syringes. The connector is connected to the catheter introduced in the fistula and secured. Coordination is primordial in the last phase of application since with one hand we should push the glue through the syringe into the fistula, while with the other we slowly retract the catheter tip through the fistula. To our opinion this is the most important phase since it is the only one requiring familiarization with the material. Failure to coordinate the two movements will lead to misplacement of the glue and failure of the procedure. A large drop of glue should be reserved to seal the external orifice of the fistula. Antibiotics and immobilization of the patient -in our opinion- offer nothing as post-interventional measures.

## Conclusion

The best treatment for a disease is the one that gives at least the same result with the others with minimised risk for the life of the patient and minimal application effort. Conservative closure of fistula with fibrin tissue sealant is simple and especially in recurrent and difficult cases. Although anal fistula surgery is associated with considerable morbidity, mainly related to anal incontinence, our patient was successfully treated with the minimally invasive method by using fibrin glue, without endangering his life despite his precarious medical state. Despite the fact that not everybody believes in the effectiveness of fibrin glue application on anal fistulas, we suggest considering this solution in cases of complex fistulas at least as primary procedure in special populations such as the immunosupressed.

## Consent

Written informed consent was obtained from the patient for publication of this case report and accompanying images. A copy of the written consent is available for review by the Editor-in-Chief of this journal.

## Competing interests

The authors declare that they have no competing interests.

## Authors' contributions

TSP and DC: Analyzed and interpreted the patient data, applied the glue and drafted the manuscript. IP: Received the patient in the outpatient department and drafted the manuscript. GP: Was the radiologist implied in the case and drafted the manuscript. PK: Was the treating physician of the patient and corrected the manuscript KS and IK: Revised the article and corrected and were involved in the monitoring of the patient SP: Was responsible for the overall treatment of the patient and corrected the manuscript. All authors read and approved the final manuscript.

## Pre-publication history

The pre-publication history for this paper can be accessed here:

http://www.biomedcentral.com/1471-230X/10/18/prepub
